# The frequency of post-traumatic stress disorder symptoms in athletes with and without sports related concussion

**DOI:** 10.1186/s40169-018-0200-y

**Published:** 2018-07-23

**Authors:** Heather E. Brassil, Anthony P. Salvatore

**Affiliations:** 1 University of Louisiana, Lafayette, LA USA; 2 University of Texas, El Paso, USA

**Keywords:** Sport concussion, mTBI, Post-traumatic stress disorder (PTSD) symptoms

## Abstract

**Background:**

Purpose of this study is to investigate the presence and frequency of post-traumatic stress disorder (PTSD) symptoms in post-concussed (PC) athletes compared to a group of healthy control (HC) athletes.

**Research design and method:**

A pre and post-test group design was used to compare a post-concussed group to a matched healthy control group of athletes. An archival database which included PC (n = 62) and HC (n = 62) participants matched on age, years of education and gender who completed a test battery at baseline and post injury, The test battery was comprised of a neurocognitive assessment, self-reported symptom inventory and PTSD symptom questionnaire. Post-concussion assessment was obtained within 0–13 days post-injury.

**Results:**

PTSD symptom scores were greater in PC post injury group (*Mdn* = 0) than for the HC group (*Mdn* = 0.0), *U *= 1282.0, *p* = 0.000, *r* = 0.34. A Wilcoxon Signed-ranks test indicated that PTSD symptom scores post-injury (*Mdn* = 0) were significantly higher than pre-injury (*Mdn *= 0), *Z *= − 2.75, *p* = 0.000, *r* = 0.35. Within the PC post injury group athletes having “difficulty sleeping” was the highest reported symptom an average of 25.8% followed by “avoiding similar situations” at an average of 19.4%. “Having trouble keeping thoughts of incident out of your head” was reported at an average of 17.7% and “flashbacks” were reported at an average of 12.9%. “Nightmares” and “feeling numb and detached” were reported at an average of 8.1 and 6.5% respectively.

**Conclusion:**

Athletes who reported no PTSD symptoms prior to sports related concussion do exhibit symptoms of PTSD. Providing a PTSD symptom questionnaire may provide a more comprehensive treatment plan for PC post injury athletes who may be at risk of chronic PTSD symptoms.

## Background

Collegiate and semiprofessional athletes who sustain repeated subconcussive and concussive traumatic brain injuries may be at risk of developing post-traumatic stress disorder (PTSD) symptoms. Traumatic brain injury (TBI) and (PTSD) are reported to be strongly associated with soldiers returning from Iraq and Afghanistan with mild traumatic brain injury [1, 2]. Repeated subconcussive and concussive traumatic brain injuries leave our soldiers “shell shocked” increasing the risk of a medical diagnosis of PTSD. Given the increase risk of concussion (mTBI) in athletes the question is do they develop PTSD symptoms?

According to the 2012 consensus statement on concussion in sports [3], a sports-related concussion (SRC) is defined as: “…a brain injury and is defined as a complex pathophysiological process affecting the brain, induced by biomechanical forces” (p. 250). A concussion may occur as a result of a direct blow to the head, face, neck or the body resulting in a jarring motion to the brain [3]. A concussion does not present as a structural injury that can be seen in neuroimaging studies [3]. This injury typically results in functional neurocognitive deficiencies and increase in post concussions symptoms. Each concussion and recovery is unique to the individual [4].

The problem of SRC is not trivial. Zukerman [5] reported the epidemiology of SRC in university teams in the US from 25 sports identifying the incidence, recurrence and mechanisms of concussion from 2009–2010 to 2013–2014. The results of the study found that 1670 SRC’s were reported with 888 occurring during competitive games (53.2%) and 782 (46.8%) during practice [5]. The study concluded that there was an increase in SCR from previous reports [5] but that this might be a result of increase in reporting rather than incident. The recurrence of SRC was 1 in 11, and the leading cause of SCR was player to player contact while contact with the playing surface and equipment also contributed to the occurrence of concussion [5]. SRC continues to be a risk associated with collegiate and semi-professional sports with as many concussion going unreported [6–8].

Traumatic events such as a TBI is associated with post-traumatic stress disorder (PTSD) [1, 4, 9]. PTSD was including in a new category, trauma and stress-related disorders in the American Psychiatric Association’s *DSM*-*5* [10]. A PTSD diagnosis must include all of the eight following criteria. Criterion A states that the person was exposed to: death, threatened death, actual or threatened serious injury, or actual or threatened sexual violence in one of the following ways: directly exposure, witnessed the trauma, learned about a close relative or close friend was exposed to trauma, that indirect exposure to aversive details of the trauma usually in the course of professional duties. Criterion B states that the traumatic event is persistently re-experienced in one of the following ways: intrusive thoughts, nightmares, flashbacks, and/or emotional distress or physical reactivity after exposure to traumatic reminders. Criterion C provides evidence of avoidance of trauma-related stimuli after the trauma in one of following ways: trauma-related thoughts or feelings, and/or trauma-related reminders. Criterion D presents with negative thoughts or feelings that began or worsened after the trauma in a least two of the following ways: inability to recall key features of the trauma, overly negative thoughts and assumptions about oneself or the world, exaggerated blame of self or others for causing the trauma, negative affect, decrease interest in activities, feeling isolated and/or difficulty experiencing positive affect. Criterion E states that trauma-related arousal and reactivity that began or worsened after the trauma at least two of the following ways: irritability or aggression, risky or destructive behavior, hypervigilance, heightened startle reaction, difficulty concentrating and/or difficulty sleeping. Criterion F, G, and H states that symptoms last more than 1 month, symptoms create distress or functional impairment and symptoms are not due to medication, substance use or other illness respectively [10].

In the civilian population 1.3–3.7% prevalence of PTSD is reported over a 12-month period and 6.6–7.8% prevalence is reported over a lifetime in contrast to the military population estimates of lifetime prevalence of 18.7–37.3% [11]. More specifically 34% of veterans with mTBI met criteria for PTSD [12] while estimates of prevalence in Iraq and Afghanistan veterans who were not treated range from 5 to 20% [2]. Co-morbid PTSD and TBI cases present with overlapping symptoms [4, 13, 14] which may lead to misdiagnosis and/or under reporting of the prevalence of the coexistence of PSTD and TBI.

Severe sport injury, such as concussions, threaten an athletes’ physical, neurocognitive and psychology integrity [15–18] meeting the first criterion of PTSD as defined by the *DSM*-*V*. Studies conducted by Newcomer and Perna [16] found that injured athletes exhibited increases in intrusive thoughts as well as avoidance as compared to non-injured athletes. Horowitz et al. [19] found that the scores reported on the impact of event scales by injured athletes were similar to scores of motor vehicle accident victims. The items on the impact of event scale are commonly reported by individuals presenting with post-traumatic disorder [19]. Studies by Peck et al. [20] and Newcomer and Perna [16] suggest that post-traumatic stress may be prolonged in injured athletes and triggered when returning to the sport environment. These studies present evidence that it is possible that athletes with injuries such as a sports related concussion may be experiencing posttraumatic stress disorder symptoms as defined by the *DSM*-*V*.

A preliminary study by Marshall [21] indicated that athletes with a concussion did not report significant levels of fear, stress and anxiety while playing their sport yet they self-reported the presence of PTSD symptoms post injury. Marshall compared a large sample of athletes’ (N = 365) preseason self-report of PTSD symptoms to a group of athletes (N = 98) post-concussion. Athletes with a concussion reported a statistically significant increase in PTSD symptoms post-concussion than the large group of athletes at preseason. Athletes with a concussion however reported a reduction in PTSD symptoms over three evaluation sessions over a 30 day period post injury. These preliminary findings require further investigation with a larger sample size and control for age, gender and years of education.

The purpose of this study was to investigate the presence of PTSD symptoms in athletes with and without concussion. This study does not attempt to diagnose PTSD in athletes but focuses the investigation on the presence and frequency of athlete’s self-reported PTSD symptoms. It was hypothesized the (a) there would be no group differences on PTSD symptom scores between the PC at baseline group and the HC group, (b) there would be no group differences on immediate post-concussion assessment and cognitive test (ImPACT) post-concussion total symptom scores (PC-TSS) between the post-concussion group (PC) at baseline and a group of healthy control athletes (HC), (c) following a sports related concussion there would be a statistically significant difference between HC and PC post injury on PTSD scores, (d) there would be a statistically significant differences on the PC-TSS scores between HC and PC post injury,(e) there would be a statistically significant difference between PC at baseline and PC post injury on PTSD scores, and (f) there would be a statistically significant differences between PC at baseline and PC post injury on PC-TSS scores.

## Methods

### Participants and recruitment

The study design was a pre and post-test PC group compared to matched to a HC group utilizing data gathered on athletes from 2007 to 2016. This archival data base includes concussed athletes (PC, n = 62) and healthy control athletes HC (n = 62) matched on age, years of education and gender who completed a baseline battery of tests comprised of neurocognitive assessment, self-reported symptom inventory and post-traumatic stress disorder symptom questionnaire.

Exclusion criteria for both groups included self-reports of psychological diagnoses or psychiatric medication, ADD/ADHD, learning disability, brain surgery, substance abuse, epilepsy, seizures, and a self-reported history of three or more prior concussions. Athletes who reported a concussion within 13 months of this study were excluded. Inclusion criterion for the concussed group required the athlete to be referred by athletic trainer or physician for a sports-related concussion and completed the assessment within 14 days of the date of injury.

### Procedures and measures

All participants completed a formal, self-report measure of post-concussions symptoms and a PTSD questionnaire as part of a larger battery of measures at the University of Texas at El Paso’s Concussion Management Clinic. The ImPACT Post-Concussion Symptom Scale (PC-TSS) is designed to measure the severity of symptoms of concussion as perceived and reported by the athlete [22]. This concussion inventory includes a 7-point Likert-type scale ranging from 0 to 6, with 0 is no difficulty with symptom and ratings of 1–6 representing mild-to-severe difficulty with symptoms with 6 being the most severe. According to Lovell [22] the PCSS’s internal consistency reliability ranged from 0.88 to 0.94 across the sample of healthy high school and college students (p. 170) and an internal consistency, r = 0.93 for the concussed athlete.

The PCSS has been classified into four clusters: migraine, cognitive, sleep and neuropsychiatric symptom clusters as presented in Table 1 [23].

**Table 1:** Post-concussion symptom clusters [23]

Migraine^a^	Cognitive	Sleep	Neuropsychiatric
Headaches	Fatigue	Difficulty sleeping	More emotional
Visual problems	Mental fogginess	Sleeping more than usual	Sadness
Dizziness	Drowsiness	Sleeping less than usual	Nervousness
Sensitivity to light	Difficulty concentrating		Irritability
Sensitivity to noise	Difficulty remembering		
Nausea	Feeling slowed down cognitively		
Vomiting			
Balance problems			
Numbness or tingling			

^a^Each item is graded from 0 (asymptomatic) to 6 (severely symptomatic)

The PTSD symptom questionnaire was developed based on the symptoms reported in the DSM-V [10]. This is a paper and pencil questionnaire consisting of six questions with a simple yes or no response (Table 2).

**Table 2:** PTSD symptom questionnaire

Q1. Are you having nightmares?
Q2. Are you having flashbacks?
Q3. Are you having trouble keeping thoughts of incident out of head?
Q4. Are you feeling numb/detached?
Q5. Are you avoiding similar situations?
Q6. Are you having difficulty sleeping?

### Statistical analysis

Summary statistics, including mean and standard deviation for all continuous variables, were calculated. The measure of effect size, *r*, was calculated by dividing *Z* by the square root of N (*r *=* Z/*√N) where small effect size = 0.1; medium effect size = 0.3 and large effect size is 0.5 [21]. Frequency distributions were determined for categorical variables. Nonparametric statistical tests were used since the data was not normally distributed as indicated by a series of Shapiro–Wilk tests of normality. Spearman Rho Correlations were used to examine each group separately on the influence of hours of sleep, number of concussions, time since injury, gender, sport (collision, contact and non-contact), level of sport (collegiate and semi-professional) and the nature of the position (defensive, offensive and individual), and language the participant selected to take the ImPACT test. All analyses were performed using SPSS (version 20) and the level of significance was set a priori at *p* < 0.05.

## Results

A total of 124 athletes were selected from the files of the Concussion Management Clinic for use in the study. From this group of athletes two groups were created and were matched on gender, age, and years of education. The HC group (*n *= 62; 40 men and 22 women) ranged in age from 18 to 24 (*M *= 19.58, *SD *= 1.362), matched the PC group (*n *= 62; 40 men and 22 women) ranged in age from 18 to 24 (*M *= 19.58, *SD *= 1.362). The HC group’s years of education, excluding kindergarten, ranged from 10 to 16 (*M* = 12.94, *SD *= 1.329), matched the PC Group who ranged in years of education from 10 to 16 (*M* = 12.94, *SD *= 1.329). For hours of sleep the night prior to the testing HC (*n *= 58) ranged from 3.5 to 10 h (*M *= 6.91, *SD* = 1.33) compared to the PC (*n *= 60) ranged from 5 to 12 h (*M *= 7.80, *SD* = 1.62). The HC group height (*n *= 60) and weight (n = 62) ranged from 59 to 79 in. (*M *= 70.85, *SD* 4.48) and 117–330 lbs. (*M* = 189.77, *SD* = 49.41) compared to the PC group (n = 62 for both measures) ranged from 60 to 81 in. (*M* = 70.40, *SD* = 4.75) and 108–338 lbs. (*M* = 186.00, *SD* = 53.38). Within the HC group (n-62) 79% reported being mono-lingual with 95.2% choosing to be tested in their first language compared to the PC (n = 62) participants where 83.9% reported being monolingual with 98.4% choosing to be tested in their first language. The time between injury and the concussion assessment for the PC group ranged from 0 to 13 days (*M *= 3.73, *SD* = 3.0) with 66.1% within 0–3 days, 21% within 4–6 days and 12.9% within 7–12 days of the injury. Table 3 provides additional demographic comparisons of the HC group and the PC group as it relates to country of origin, type of sport, the level of sport, nature of the position played in that sport and handiness. Table 4 provides various frequencies of characteristics as it relates to the number of concussions at the time of testing. Table 5 provides the ImPACT composite scores for the PC and HC groups.

**Table 3:** Demographic of the healthy controls and post-concussed groups N = 124

	Healthy controls (HC)		Post-concussed (PC)	
	*n *= 62		*n *= 62	
	Frequency	Percent	Frequency	Percent
Country of origin				
USA	56	90.3	58	93.5
Canada	1	1.6	0	0.0
Mexico	2	3.2	1	1.6
Latvia	2	3.2	0	0.0
Czech Republic	0	0.0	1	1.6
Belgium	0	0.0	1	1.6
Slovakia	1	1.6	0	0
Puerto Rico	0	0.0	1	1.6
Sport				
Collision	35	56.5	34	54.8
Contact	23	37.1	26	41.9
Non-contact	4	6.5	2	3.2
Type of sports				
Football	28	45.2	25	40.3
Ice hockey	7	11.3	9	14.5
Soccer	1	1.6	5	8.1
Basketball	9	14.5	7	11.3
Softball	9	14.5	7	11.3
Baseball	1	1.6	4	6.5
Volleyball	3	4.8	3	4.8
Cheerleading	4	6.5	2	3.2
Level of sport				
Collegiate	56	90.3	53	85.5
Semi professional	6	9.7	9	14.5
Nature of position				
Defensive	26	41.9	22	35.5
Offensive	31	50.0	38	61.3
Individual sport	5	8.1	2	3.2
Handiness				
Right	53	85.5	52	83.9
Left	4	6.5	7	11.3
Ambidextrous	5	8.1	3	4.8

**Table 4:** Frequencies of characteristics of history of concussion and games missed due to concussion

No. of concussions	Healthy controls		Post-concussion	
	n = 62		n = 62	
	Frequency	Percent	Frequency	Percent
Prior to assessment				
0	48	77.4	30	48.4
1	10	16.1	23	37.1
2	4	6.5	9	14.5
With loss of consciousness				
0	60	96.8	50	80.6
1	2	3.2	11	17.7
2	0	0.0	1	1.6
Resulted in confusion				
0	56	90.3	43	69.4
1	6	9.7	15	24.2
2	0	0.0	4	6.5
Difficulty remembering events immediately after concussion				
0	59	95.2	51	82.3
1	2	3.2	10	16.1
2	1	1.6	1	1.6
Difficulty remembering events that occurred				
0	59	95.2	53	85.5
1	3	4.8	6	9.7
2	0	0.0	3	4.8
Total games missed				
0	56	90.3	50	80.6
1–3	5	8.0	6	9.7
4–6	1	1.6	3	4.8
7–12	0	0.0	3	4.8

**Table 5:** Immediate post-concussion assessment and cognitive test (ImPACT) composite scores N = 124

	PC at baseline		PC at post-injury		HC	
	*n *= 62		*n *= 62		*n *=62	
	M	SD	M	SD	M	SD
Verbal memory	84.66	11.00	82.92	14.61	86.0	10.24
Visual memory	73.65	13.18	70.05	15.29	76.37	13.86
Vis. motor speed	38.14	6.40	37.69	7.24	38.74	6.24
Reaction time	0.60	0.07	0.60	0.10	0.60	0.07
Impulse control	4.19	2.41	5.21	4.37	4.54	2.80

### Statistical analysis of demographic variables

There were no statistically significant differences between the healthy control (HC) group and the post concussed (PC) post injury group based on gender, age, and years of education. There were statistically significant differences on the hours of sleep between the PC post injury group (*M *= 7.8, *SD* = 1.62) and HC group (M=6.91, *SD* = 6.91) and the number of concussions between the PC post injury group (*Mdn* = 1) and HC group (*Mdn* = 0.0), *U* = 1365.00, *p* = 0.001, *d* = 0.29; small effect). The influence of hours of sleep and the number of concussion on the scores of PTSD and post-concussion symptom scores (PC-TSS) were examined for each group using Spearman’s rho correlation. There was no significant relationship based on number of concussions or hours of sleep on PTSD symptoms or PC-TSS scores. Additionally, there were no relationships found for gender, sport (collision, contact and non-contact), level of sport (collegiate and semi-professional) and the nature of the position (defensive, offensive and individual). The influence of time since injury was examined within the PC post injury group on PTSD symptoms which also showed no significant relationship. As a result these findings these variables were not considered in further analysis.

### Comparison of PTSD scores between concussed (PC) at baseline and healthy control (HC) groups

Mann–Whitney-*U* tests were conducted to investigate the difference in PTSD symptom scores between the PC group at baseline and HC group. As expected there were no statistically significant differences between the groups for the PTSD symptom scores. The frequencies for each of the PTSD questions are presented in Table 6 and the descriptive statistics, Mann–Whitney U-tests, effect sizes (d) and significance levels are presented in Table 6.

**Table 6:** Frequency for each of PTSD questions

	PC at baseline		PC		HC	
	N = 62		N = 62		N = 62	
	Yes	No	Yes	No	Yes	No
Are you having nightmares?	7	55	5	57	1	61
Are you having flashbacks?	6	56	8	54	7	55
Are you having trouble keeping thoughts of incident out of your head?	4	58	11	51	1	61
Are you feeling numb/detached?	2	60	4	58	0	62
Are you avoiding similar situations?	2	60	12	50	5	57
Are you having difficulty sleeping?	6	56	16	46	3	59

Yes and no responses

### PC-TSS scores between concussed (PC) at baseline and healthy control (HC) groups

It was hypothesized that there would be no group differences in the PC group at baseline and the HC group on PC-TSS cluster scores. Mann–Whitney-*U* tests were conducted to investigate the difference in PC-TSS cluster scores between the PC group at baseline and HC group. As expected there were no statistically significant differences between the groups on the PCSS scores. Descriptive statistics, Mann–Whitney U-tests, effect sizes (d) and significance levels are presented in Table 7 for the group comparisons.

**Table 7:** ImPACT total symptom score cluster descriptive statistics, Mann–Whitney U-tests, effect sizes (d) and significance levels (p) N = 124

	PC at baseline		HC		PC at baseline vs HC		
	*n *= 62		*n *= 62				
	M	SD	M	SD	Mann–Whitney U	*r* ^a^	*p*
Total PTSD	0.09	0.391	0.18	0.43	1553.50	0.17	0.08
Total PC-TSS	3.57	4.02	2.83	4.26	1541.00	0.13	0.16
Migraine cluster	1.24	2.51	0.63	1.72	1659.00	0.15	0.11
Cognitive cluster	1.06	1.77	1.29	2.42	1916.50	0.00	0.98
Sleep cluster	1.44	2.12	1.00	1.75	1696.00	0.12	0.20
NeuroPsy cluster	0.66	1.61	0.48	1.49	1848.00	0.05	0.60

*PC at baseline* baseline pre-injury in PC group, *HC* healthy control, *M* mean, *SD* standard deviation, *r* effect size, *p* Asymp. Sig. (2 tailed)

^a^Small effect size = 0.1; medium effect size = 0.3 and large effect size is 0.5 [21]

### Comparison of PTSD scores between concussed (PC) post injury and healthy control (HC) groups

As expected the PC group post injury PTSD symptom scores were significantly higher compared to the HC group. The PC group post injury PTSD symptom scores were greater (*Mdn* = 0.00) than for the HC group (*Mdn* = 1.0), *U *= 1282.00, *p* = 0.000, *r* = 0.34 (medium effect).

### PC-TSS scores between concussed (PC) post injury and healthy control (HC) groups

It was hypothesized that following a SRC there would be statistically significant differences in PC-TSS scores between the PC post-injury and HC groups. Descriptive statistics, Mann–Whitney *U* test (due to non-normal distributions) and effects sizes [24] for the post-concussion symptom scores are presented in Table 8.

**Table 8:** Descriptive statistics for both groups are presented below; Mann–Whitney U-tests, effect sizes (d) and significance levels (p) N = 124

	PC-post injury		HC		PC post injury vs HC		
	*n *= 62		*n *= 62				
	M	SD	M	SD	Mann–Whitney U	*r* ^a^	*p*
Total PTSD	0.90	1.28	0.18	0.43	1282.00	0.34	0.000
Total PC-TSS	19.52	19.51	2.83	4.26	613.00	0.58	0.000
Migraine cluster	8.52	9.07	0.63	1.72	554.00	0.65	0.000
Cognitive cluster	6.76	6.98	1.29	2.42	909.00	0.48	0.000
Sleep cluster	2.37	3.12	1.00	1.75	1458.00	0.23	0.000
NeuroPsy cluster	1.76	3.56	0.48	1.49	1529.50	0.22	0.000

*PC* post concussion, *HC* healthy control, *M* mean, *SD* standard deviation, *r* effect size, *p* Asymp. Sig. (2 tailed)

^a^Small effect size = 0.1; medium effect size = 0.3 and large effect size is 0.5 [21]

As expected the PC group post injury symptom scores were significantly higher for PC-TSS compared to the HC group scores. The PC group post injury PC-TSS scores were greater (*Mdn* = 13.50) than for the HC group (*Mdn* = 1.0), *U *= 613.00, *p* = 0.000, *r* = 0.58 (large effect). The PC post injury Migraine Cluster scores were greater (*Mdn* = 6.0) than for the HC group (*Mdn* = 0.00), *U *= 554.0, *p* = 0.000, *r* = 0.65 (large effect); Cognitive Cluster scores were greater in the PC post injury (*Mdn* = 5.0) than for the HC group (*Mdn* = 0.00), *U *= 909.00, *p* = 0.000, *r* = 0.48 (medium to large effect); Sleep Cluster scores were greater in the PC post injury (*Mdn* = 1.0) than for the HC group (*Mdn* = 0.00), *U *= 1458.0, *p* = 0.000, *r* = 0.23 (a small to medium effect; and NeuroPsy Cluster scores were greater in the PC post injury (*Mdn* = 0.00) than for the HC group (*Mdn* = 0.00), *U *= 1529.50, *p* = 0.000, *r* = 0.22 (small to medium effect).

### Comparison of PTSD scores at baseline and post injury for the PC group

A Wilcoxon Signed ranks test indicated that PTSD symptom scores post-injury (*Mdn* = 0) were statistically significantly higher than pre-injury (*Mdn *= 0), *Z *= − 2.75 *p* = 0.006, *r* = 0.35 (medium effect). Three of the six questions were statistically significantly higher after injury: Having trouble keeping thoughts of incident out of your head, *Z *= − 2.11, *p* = 0.035, *r* = 0.35 (medium effect); avoiding similar situation, Z = − 2.673, *p* = 0.008, *r* = 0.34 (medium effect), and having difficulty sleeping, *Z* = − 2.50, *p* = 0.012, *r* = 0.32 (medium effect). Within the PC post injury group “having difficulty sleeping” was the highest reported symptom at 25.8% followed by “avoiding similar situations” reported at 19.4%. “Having trouble keeping thoughts of incident out of your head” at 17.7% and “flashbacks” were reported at 12.9%. The frequency of “nightmares” and “feeling numb and detached” were reported at 8.1 and 6.5% respectively.

### Comparison of PC-TSS scores for the PC group at baseline and post injury

A Wilcoxon Signed-ranks test indicated that PC-TSS scores post-injury (*Mdn *= 13.5) were significantly higher than at baseline (*Mdn *= 0), *Z *= − 5.631, *p* = 0.000, *r* = 0.72 (large effect). Post-injury migraine cluster scores (*Mdn *= 6.0) were significantly higher than pre-injury (*Mdn *= 0), *Z *= − 1.953, *p* = 0.051, *r* = 0.25 (small to medium effect); cognitive cluster post injury scores were statistically greater in the PC post injury (*Mdn* = 5.0) than for the PC at baseline (*Mdn* = 0.00), *Z *= − 5.378, *p* = 0.000, *r* = 0.68 (large effect); Sleep Cluster post injury scores were statistically greater in the PC post injury (*Mdn* = 1.0) than for the PC at baseline (*Mdn* = 0.00), *Z *= − 2.961, *p* = 0.003, *r* = 0.38 (medium effect); and NeuroPsy cluster scores were greater in the PC post injury (*Mdn* = 0.00) than for the PC at baseline (*Mdn* = 0.00), *Z *= − 2.604, *p* = 0.009, *r* = 0.33 (medium effect).

## Discussion

The literature is rich with discussions of the comorbidity of PTSD and mTBI with soldiers returning from Operation Enduring Freedom and Operation Iraqi Freedom [1, 12, 19]. However little is known about PTSD symptoms in athletes who have experienced a SRC. Given the increase risk of concussion (mTBI) in athletes the question is do they develop PTSD-type symptoms? To our knowledge, this is one of the first published study’s to compare healthy athletes to concussed athletes matched on age, gender, years of education who were assessed in the early stages of a SRC. Specifically, a pre and post injury PTSD symptoms questionnaire was administered to a group of concussed collegiate athletes. The findings of this study indicated that: PC athletes reported greater incidence of PTSD symptoms compared to HC athletes and PTSD symptoms were greater post-injury when compared to baseline results in the PC athletes.

King [20] suggested that PCSS and PTSD symptoms can and do co-occur in individuals with mild traumatic brain injury. Results from our study indicated that PC and HC athlete’s scores of PC-TS0S and PTSD symptoms were significantly different between the groups. PC-TSS symptom scores of concussed athletes within 13 days of injury were found to be high (*M *= 19.52, *SD *= 19.51) [15]. Furthermore PTSD symptoms were present and significantly higher in the PC group post injury when compared to HC. Additionally the pre and post injury PC group’s TSS and PTSD symptoms were significantly different. Finally, The PC TSS clusters (migraine, cognitive, sleep and neuropsy) and PTSD symptoms were all found to be statistically higher at post injury.

Vanderploeg et al. [22] found statistically significant increases in PTSD symptoms when comparing service personnel with a single TBI to those with multiple TBI’s. This is contrary to the findings of this study which focused on SRC. In the present study there was no statistically significant relationship between the number of concussions on PC-TSS and PTSD symptom scores. It should be noted that the sample in Vanderploeg study was significantly larger than the sample in the present study. Further studies in SRC with larger sample sizes may better address the interaction of history of multiple concussions and the reporting of PTSD symptoms.

Peskind el al [4] indicate that the nature of the environment in which the brain injury occurs contributes to the onset or presence of PTSD symptoms. Meyer, Jaffee and Grime [23] state that soldiers are under great psychological stress while civilian injuries occur in relatively controlled environment with minimal stress and anxiety. A preliminary study by Marshall [21] indicated that athletes with concussions did not report significant levels of fear, stress and anxiety yet self-reported PTSD type symptoms were present. The findings of the current study provide further evidence that athletes with SRC report elevated PTSD symptoms in the absence of a heightened fearful environment. To the contrary, athletes were participating in a sport they enjoyed and played in an atmosphere of heightened excitement. Still PC athletes reported “having difficulty sleeping” at 25.8% followed by “avoiding similar situations” at 19.4%. “Having trouble keeping thoughts of incident out of your head” was reported 17.7% and “flashbacks” were reported at 12.9%. Nightmares and feeling numb and detached were at 8.1 and 6.5% respectively. Further analysis of the frequency of specific PTSD symptom questions found that recurring thoughts of the incident, avoidance and sleep were all statistically significantly higher post injury. Compared to military personnel that suffer with PTSD after mTBI, the athletes experienced a concussion during a sports activity that they enjoy, present with PTSD symptoms in SRC While it is unknown whether the athletes experienced fear or anxiety in the event one would assume they did not. However, after experiencing the cognitive, visual, auditory, emotional dysfunction following a concussion they may have experienced such fear and anxiety associated with the injury after the fact.

### Limitations

There are several limitations within this study. Some data was collected in a group setting and some in a one-on-one setting over a period of 8 years. Therefore, there may be some impact on the results given the different test environment All of the participants that were assessed and tested in the same clinic, with participants all coming from the same small geographic area potentially limiting the generalization of the results. All PC evaluations where conducted within 14 days of each athlete’s SRC.

### Clinical implications

In this study the symptoms of PTSD and PC-TSS were found to co-occur with a SRC. Clinicians should consider the following: it can be anticipated that the PTSD symptoms may be present and may resolve, however, an awareness that the PTSD symptoms may persist if athlete develops post-concussion syndrome. A multidisciplinary approach with PTSD symptomatology may be a reasonable approach for SRC.

### Future research

Further research is recommended to explore the degree of severity of PTSD symptoms reported, and the degree of fear, anxiety and stress associated with the prevalence of PTSD symptoms over time. Utilization of a multidisciplinary research team would enhance the study of the comorbidity of PTSD symptoms and SRC. The use of a PTSD assessment tool with appropriate psychometric properties will strengthen studies in the future. A longitudinal study that provides a comparison of multiple causes of injury i.e. civilians: car accidents, domestic violence, rape, violent acts, athletes with SRC, and combat soldiers (blast vs non blast) would allow for a more comprehensive presentation of the co-occurrence of PTSD and mTBI.

## Conclusion

This is the first published articles that investigated the presence of PTSD symptoms reported in athletes with a SRC. Athletes in the early stages of concussion self-reported PTSD symptoms. These results suggest that there is a potential to use a PTSD symptom screening tool to assess the comorbidity of concussion and PTSD. A multidisciplinary approach with PTSD symptomatology may be a reasonable approach for sports related concussion.
